# Scaling in the space-time of the Internet

**DOI:** 10.1038/s41598-019-46208-6

**Published:** 2019-07-05

**Authors:** István Papp, Levente Varga, Mounir Afifi, István Gere, Zoltán Néda

**Affiliations:** Babeş-Bolyai University, Department of Physics, str. Kogalniceanu 1, Cluj-Napoca, Romania

**Keywords:** Computer science, Statistics

## Abstract

The Internet on the router level, is a complex network embedded in a geographical space. We provide experimental evidences suggesting that the average travel time for a message, with fixed length, increases roughly as the square root of the geographical distance. To understand this scaling law and other measurable topological properties of the Internet as a graph, we introduce and study a simple network model. The model is based on a few realistic socio-economic facts/assumptions and qualitatively reproduces the experimentally observed stylized facts.

## Introduction

The Internet’s topological structure is in the continuous focus of network and computer scientists^1,2^. In contrast with similar large communication networks, like the wired telephone network for example, which grew according to a designed topology governed by a central administration, the Internet grows in a less controlled manner. This rapid and seemingly uncontrolled growth raises a lot of problems with routing, resource reservation and administration. In order to keep track of the potential problems and offer rapid solutions to these, one has to understand the topological structure of the Internet as a graph embedded in a geographical space. Right from the early days of the Internet it was clear that beside gathering useful experimental facts on the properties of the Internet, modelling is also necessary. In the absence of large-scale topological data initial studies focused on simple heuristic models. As a first attempt simple random growth was considered as the main driving process^3,4^. Later, when experimental data suggested that we deal with a small-world type network that exhibits a “fat tail” degree distribution, models that used random but preferential growth were considered^1^.

As more accurate measurements on the topology of the Internet became possible, the preferential attachment driven models were improved^5,6^ in order to explain more subtle topological features as well. Models based on first principles, starting from the main question “what really matters when it comes to topology construction?” were also considered more than a decade ago, and these approaches explained also the relative robustness of the Internet against targeted attacks on the hubs^7,8^.

More research went in the direction of k-shell decomposition models^9,10^, and location based growth^11,12^. Considering the Internet as a spatial network (a network embedded in the geographical space)^13^, was also helpful in understanding it’s evolution and making connections with important socio-economic parameters^1,12,14^. Topological features of the Internet are correlated with the data transmission speed, and as a result many useful statistical information concerning the topology can be extracted from Round Trip Time (RTT) measurements with “ping” or “traceroute” commands^15,16^.

In studying large complex system like the Internet, a first task is to reveal universally valid statistical features and laws. Statistical physics taught us, that complexity is always accompanied by scaling laws^17^, i.e. power law type dependence among relevant variables. In many case scaling is just a first, simpler approach for a more complicated functional dependence, however scaling can have also a deeper meaning suggesting universalities and self-organisation. Proving that a functional dependence that resembles a power-law is indeed a rigorous scaling with deeper consequences is not a trivial task and needs a more elaborated statistical and modelling study. In our approach we will look for scaling laws only as a first, simpler approach to the relevant functional dependences in the observed trends.

Recently our group revealed an interesting dynamical scaling between travel time and travel distance (measured along the geodesic lines), holding on ten orders of spatial magnitudes for human traveling modes^18^. The coarse-grained result is that the estimated average travel time scales roughly proportionally with the square root of the travel distance. We argued that this interesting scaling law appears partly due to the topology of the complex network on which mobilities are made and partly due to some socio-economic effects (crowding, speed restrictions, etc.). Our aim here is to prove the existence of a similar dynamical scaling for the RTT on the Internet and understand it in the framework of a simple and realistic network model embedded in the geographical space. The Internet model we are attempting to construct here will be motivated by simple first-principle socio-economic assumptions. The model is able to reproduce statistically the known topological properties that can be measured by large scale “traceroute” experiments^15^. The present work offers thus contribution in this research field from two direction. First, as an experimental result we present an interesting and universally valid statistical law for the data transmission speed on the Internet. Second, we consider a simple modelling approach to the Internet as a graph, trying to understand in a unified context all stylised facts (including the one reported here) known for the Internet.

The present study does not aim at ending the debates on the Internet’s topological properties nor at giving solutions related to its’ rapid growth. We only intend to emphasise another stylised fact and a simple explanation for it, that might have had less attention recently and could be of importance in future engineering and optimisation problems for the Internet.

## Average Round Trip Time as a Function of Distance

**Figure 1:**
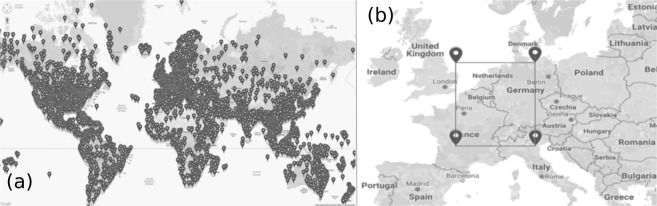
Experiments for “ping” and “traceroute”. (**a**) Target locations of the “ping” experiment. (**b**) Restricted area for the “traceroute” experiment. The Figures were generated by using the version 3.11 of the Google Maps JavaScript API (Application Program Interface).

We consider both a large-scale “ping” experiment and use also the freely available results of the CAIDA UCSD IPv4 Routed/24 Topology Dataset^15^ obtained with “traceroute”. These experiments prove that the average response time (RTT) is increasing with the square root of the distance (measured on geodesic lines) between the considered routers. The Caida dataset offers also information on the topology of the Internet as a graph. The “ping” experiments were conducted around the globe starting the command from two fixed locations in Europe (see targets on Fig. 1a), while the “traceroute” measurements aimed to reveal also the network topology, is restricted to a well-delimited area of Europe (see locations on Fig. 1b).

### Ping experiment

The experiment is based on the Internet Control Message Protocol (ICMP) echo request packet sending and receiving^19^. We relied on the “ping” command^20^, which is used on most platforms to test the ability of a source computer to reach a specified destination computer. It operates by sending an operating system based ICMP echo request to a specified IP address, receiving also the response time elapsed from sending the request and getting back the echo from the destination. This time is usually measured in *ms*. In total 24700 destination computers were selected from various locations on the Earth (see Fig. 1a). Their geographical coordinates (GPS coordinates) were determined from the IP addresses using the home page: IP2LOCATION^21^. Two source locations (from where the ping command were generated) were considered: one in Budapest (Hungary) and one in Cluj-Napoca (Romania). By using the GPS coordinates we calculated the geodesic distance (*d*) between the source and target routers.

**Figure 2:**
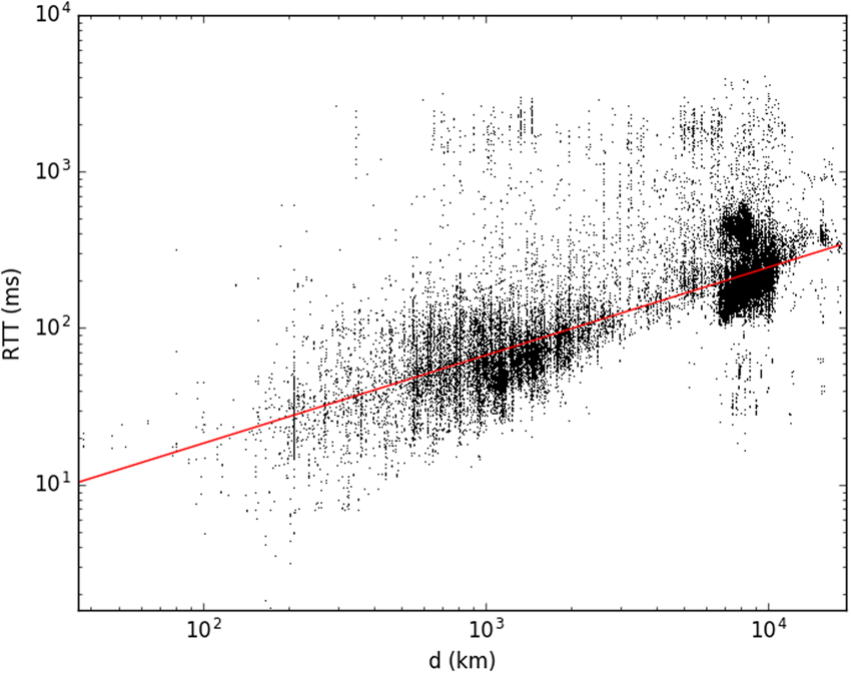
Raw data points from measurements, the black dots represent an average response time in *ms* (RTT) for one destination address. The continuous red line indicates a power-law trend with exponent 1/2. Please note the logarithmic scales.

**Figure 3:**
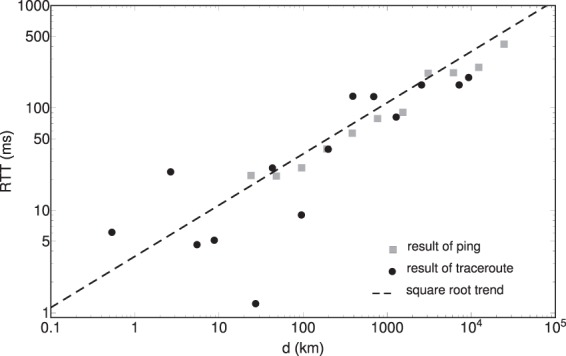
Averaged round trip time of the ping and traceroute experiments using a logarithmic binning method. The dashed line indicates a power-law trend with exponent 1/2.

The destination routers’ ability to respond was tested for weeks on a 24 hour basis. Response times were averaged on the number of times the target was reached. Experiments were conducted between September 2013 and March 2015. On Fig. 2 we plot the averaged RTT values against the distance *d*, for all pinged router pairs. Using a logarithmic binning method (exponentially increasing bin-sizes) one gets the averaged trend plotted with black dots on Fig. 3. From Figs 2 and 3 we draw the first qualitative conclusion that the response time scales roughly as ${\rm{RTT}}\propto \sqrt{d}$.

### Traceroute experiment

“Traceroute” operates in a somehow similar manner as “ping” does. In this case the ICMP echo request maps the RTT round trip time, but with the difference that it repeats itself with increasing life time (or TTL - time to live) settings, revealing the intermediate hops as well. This process roughly follows the steps: (1) the sender sends out a sequence of three datagrams to an invalid port address with a TTL set to 1; (2) the first router to receive the packets will send it back with TTL set to 0; (3) the sender now will send the message again with TTL set to 2; (4) the packet will be received back now after two hops; (5…) the process is repeated incrementing TTL with unity until the message reaches its final destination; (n) since the packages were sent to an inexistent port the final destination replies with an ICMP Port Unreachable message.

For this experiment we used the already available data from the IPv4 Routed/24 Topology Dataset^15^, which contains summaries of “traceroute” probing with the Paris Traceroute protocol since the year 2007^15^. Our aim was to use recent data, so we limited our study to files only from 2017. The traceroute dataset allows to reveal also the network topology on router level, since we can map all first-order neighbours of the nodes (routers in our case). The most active monitors at that time were from Netherlands and Switzerland. As a consequence, for an easier handling of the large dataset and to reduce the computational challenges with the big amount of data, we selected a restricted geographic area sketched in Fig. 1b on which we built a subnetwork of the Internet at router level. The giant component of this subnetwork contained ${N}_{g}=43992$ nodes and ${L}_{g}=191005$ internal links.

Before focusing on the unveiled graph topology and compare the observed features with the ones given by models, we present here our results for the RTT versus distance (*d*) scaling, as it is revealed by these experiments. In this case the source for the traceroute experiment were located inside the region shown in Fig. 1b and the target routers were spread all over the World. On Fig. 3 we plot together the logarithmically binned data^18^ for the “ping” and “traceroute” experiments. Apart of the fact that the data for the traceroute experiments scatter in a larger manner due to a poorer statistics, both data is consistent with a power law trend having the scaling exponent of 1/2.

Considering a fit of the experimental results in the form of ${\rm{RTT}}=a\cdot {d}^{1/2}$ the obtained *R*^2^ coefficients of determination were ${R}^{2}=0.98$ for the ping data and ${R}^{2}=0.88$ for the traceroute data. In such a view the assumed scaling can be considered also quantitatively acceptable.

## Model

In order to explain the non-trivial scaling found in the previous section we consider a network model based on a simple wiring rule. We will argue in the next section that the model is successful not only in reproducing the RTT versus distance scaling, but it is successfull also in generating the right network topology.

Within the model the nodes of the graph represent cities while links are wiring channels between them (network cables). In the simplest approximation *N* nodes are uniformly distributed in the Euclidean space. The considered territory is a square with edges of unit size.

**Figure 4:**
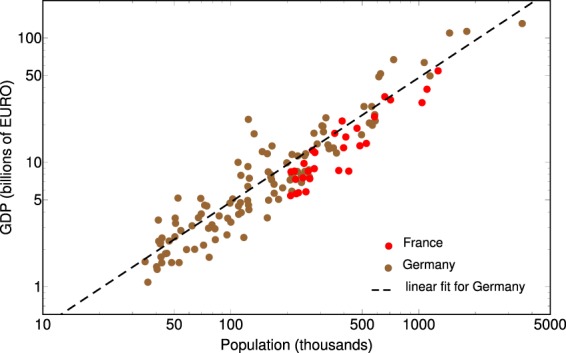
Linear proportionality between the GDP and population for some large cities in Germany and France.

Recent results indicate that the “connectivity” of a city usually depends on it’s Gross Domestic Product (GDP)^22,23^. An equivalent formulation of this is that the probability for a person in the given city to be “wired” depends on the GDP per capita. In the studied region however, the city’s GDP is roughly proportional with the total population, since we considered a territory where the economic development is rather homogeneous. In Fig. 4, we illustrate this proportionality for some large cities in Germany and France. Data for Germany are taken from the Internet^24^, while the data for France is obtained form a discussion forum on the Internet. The linear fit for the cities in Germany has a coefficient of determination ${R}^{2}=0.89$, which is a reasonable value for admitting our hypothesis.

In such a view the population of the cities (*W*_*i*_) will determine the weight (${\omega }_{i}$) of the nodes used in the wiring process (see later), and we consider the *W*_*i*_ values distributed according to a Tsallis-Pareto type distribution with exponent $\alpha =1$. Such city-size distribution is realistic for not too small settlements (in general *W*_*i*_ > 10^3^ − 10^4^ inhabitants) and is confirmed in many geographical areas^25,26^. For smaller settlements a better statistics is given by the log-normal distribution^25,26^, taking into account however that the wired Internet network interconnects mainly the larger cities, we consider that the Tsallis-Pareto distribution is justified in our model. For generating the Tsallis-Pareto distribution of the *W*_*i*_ values we used the implemented generator from the *scipy* package of Python^27^. In order to simplify the scaling properties of our model the generated *W*_*i*_ values were renormalized, so that the maximal value to be always one.

We assume the relation between *W*_*i*_ and ${\omega }_{i}$ in the form: ${\omega }_{i}=\beta \sqrt{{W}_{i}}$. The values of ${\omega }_{i}$ are defining the “radius of connectivity” for each settlement. The “wealth or GDP” of each city is assumed to be proportional with its population, therefore we assume that the area around it that can be supported with links is proportional with *W*_*i*_. This leads us to the considered hypothesis: it’s “radius of connectivity” should be proportional with the square-root of *W*_*i*_. In the proposed kernel *β* is a proportionality factor.

**Figure 5:**
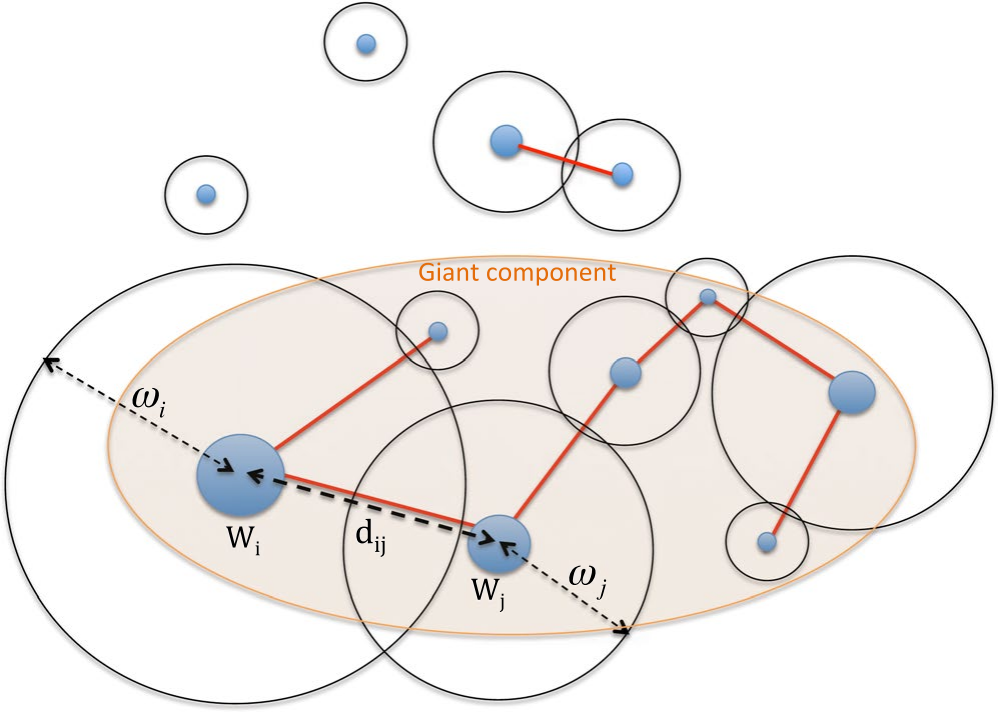
Main elements of the model and the connection rule.

For wiring the nodes first we determine for each pair of nodes the

1 $${f}_{ij}=\frac{{\omega }_{i}+{\omega }_{j}}{{d}_{ij}}$$

fraction, where *d*_*ij*_ is the geodesic distance between the two cities. In case ${f}_{ij} > 1$ we connect the two nodes, otherwise they remain unconnected. This wiring condition models the obvious fact that the total wealth of the cities govern their socio-economic ability to build a direct link between them. Small cities at large distances are unlikely to be connected, while large cities at short distances become connected. Also, when connecting two settlement one has to take contribution from both by adding their radius of connectivity. The main elements of the model are sketched in Fig. 5.

The model in it’s actual form has two parameters: *N* and *β*. Changing the value of *N* affects the density of the nodes and implicitly the average distance between them. However, due to the rescaling of the *W*_*i*_ values, the imposed Pareto-type distribution and the connectivity condition one might expect that the statistical properties of the graph will not be seriously affected by changing *N*. This guess has to be however proved by computer simulations. In contrast with the parameter *N*, we expect that the *β* parameter influences strongly the obtained graphs giant component.

Simulations for a relatively large system ($N=2400$ and $N=8000$) and several *β* values were carried out. We searched for the best value of *β* that is able to reproduce the experimentally observed scaling laws and the average number of links/node (8.68) for the giant component. For each parameter set the computer simulation results were averaged over 100 independent realizations of the graph with fixed *N* and *β* parameters.

## Model versus Reality. Results and Discussion

In order to validate the previously introduced model we compare the statistical properties of the network unveiled by the traceroute experiments and the one generated by the model. The main advantage of trace-route experiments is that one can reveal also the routers on which the information passed. By this one can reconstruct the links in the studied network.

### Network topology

**Figure 6:**
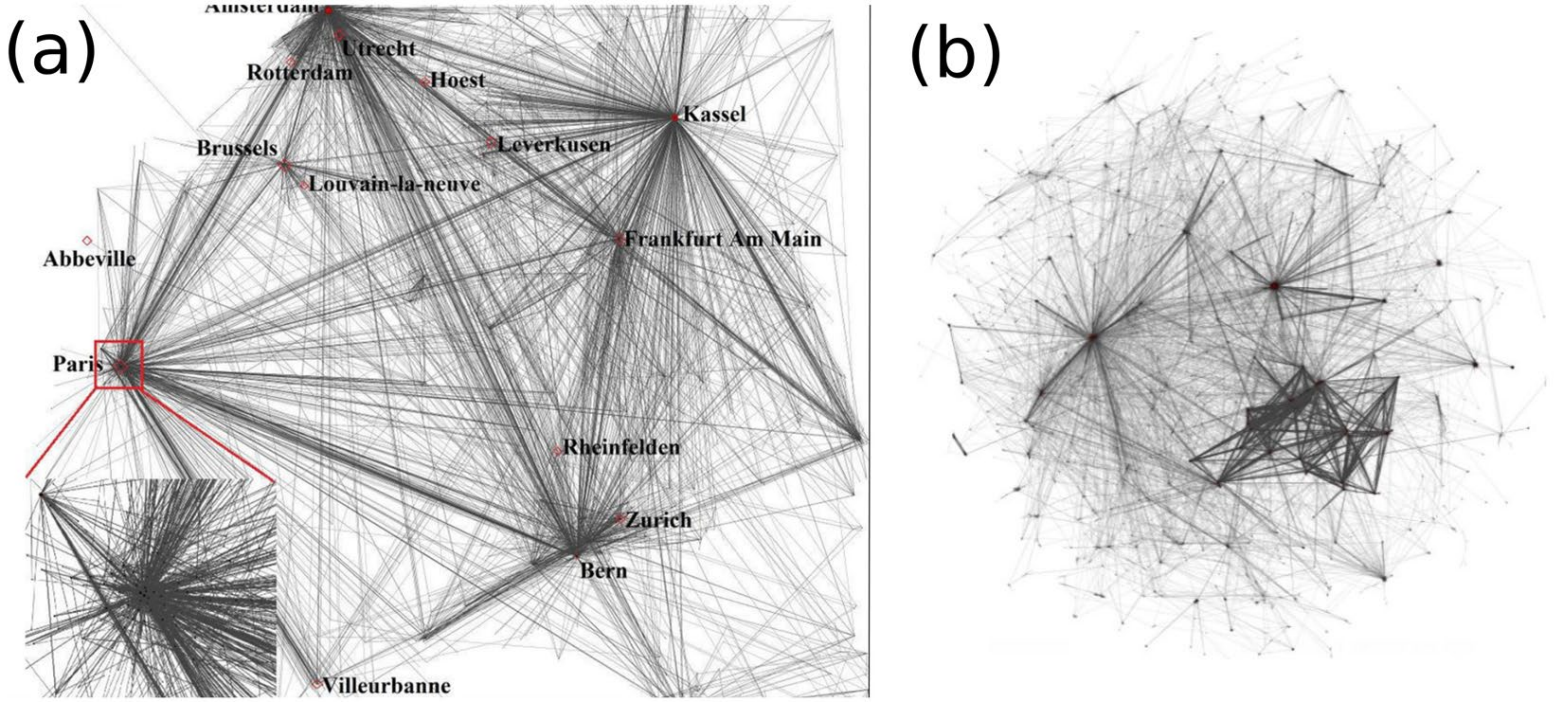
Network structure unveiled from traceroute experiments. (**a**) The largest component of the graph for the studied region. The Internet at router level embedded in the real geographical space with a zoom in the Paris region. (**b**) The Fruchterman-Reingold layout of the graph, constructed with the Gephi drawing application^32^.

**Figure 7:**
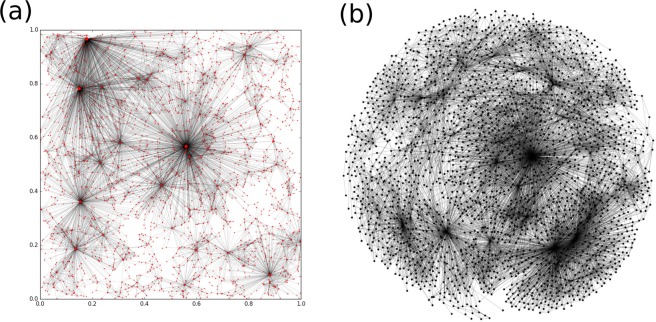
Network structure given by the model for $N=2400$ and $\beta =0.4$ (${N}_{g}=2380$, ${L}_{g}=12809$). (**a**) The graph generated by the model and embedded in the geometrical space ((0, 1) × (0, 1)). (**b**) The Fruchterman-Reingold layout of the generated graph.

The largest component of the experimentally studied network plotted in the real geographical space is shown in Fig. 6a. The topological representation of the largest component of this graph with the Fruchterman-Reingold layout is given on Fig. 6b. For $N=2400$ we obtained that the experimentally observed features are best reproduced by the model if one considers $\beta \approx 0.4$. A realization of the networks giant component generated with these parameters is shown in Fig. 7. In agreement with our initial guess, we found that changing the value of *N* does not affect in a considerable manner the giant components statistical properties. Simulation for $N=8000$ and $\beta =0.4$ yields giant components with statistically similar graph characteristics. Some properties are summarized and compared in Table 1.

**Table 1:** Characteristic network quantities compared with the measurements given by different methods^29^.

	SKITTER from^29^	BGP from^29^	WHOOIS from^29^	traceroute this work	model N = 2400	model N = 8000
〈*N*_*g*_〉	9204	17446	7485	43992	1946	6679
〈*L*_*g*_〉	28959	40805	56949	191005	12607	49748
〈*k*〉	6.29	4.68	15.22	8.61	12.95	14.88
〈*k*_*max*_〉	2070	2498	1079	10474	1382	4254
*α*	1.25	1.16	—	1.23	1.52	1.60

The modeling values are results averaged over 100 runs.

The average degree of the nodes mapped in our subnetwork is $\langle k\rangle =8.68$. The degree-distribution for the largest component is fitted with a Paretto-Tsallis (or Lomax II) distribution:

2 $$p(k)=\frac{\alpha }{(\alpha -1)\,\langle k\rangle }{(1+\frac{k}{(\alpha -1)\langle k\rangle })}^{-1-\alpha }$$

**Figure 8:**
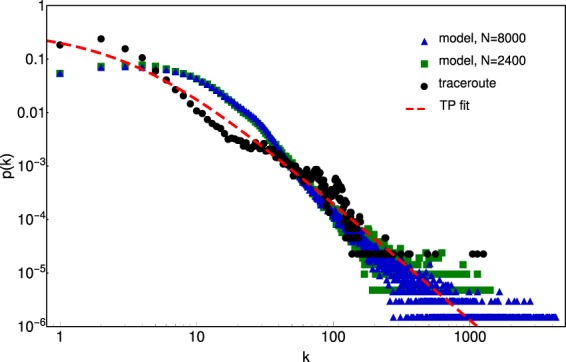
Degree distribution of the experimentally studied subnetwork (black dots) and the one generated by the model (green squares for $N=2400$ and blue triangles for $N=8000$). The dashed line indicates the fit given by the Tsallis-Pareto distribution (2) with $\alpha =1.23$ and $\langle k\rangle =8.68$. The coefficient of determination for the experimental data is ${R}^{2}=0.85$, and for the data provided by the model is always larger than ${R}^{2}=0.9$.

The experimental results obtained for this subnetworks degree distribution is plotted with black dots on Fig. 8, and the Paretto-Tsallis fit (2) with $\alpha =1.23$ is indicated with the dashed line. We recall here that such degree distributions with a scale-free tail was modelled mostly in the framework of a preferential growth model with an exponential dilution^25,28^.

One can also determine the degree distribution for the graph generated by the model. Comparison between the experimentally measured degree distribution and the one given by the model (Fig. 8) suggests a reasonable agreement (see also Table 1). In Table 1 we summarize the estimated topological properties for the Internet on router level obtained with different methods^29^, estimations from our traceroute experiments and the results of our model for $N=2400$ and $N=8000$ ($\beta =0.4$).

The obtained structural characteristics of the studied subnetwork are in agreement with previous Internet mapping measurements^29^. A more detailed presentation of the experimental results can be found in the master-thesis of Mounir Afifi^30^.

### Round trip time statistics

**Figure 9:**
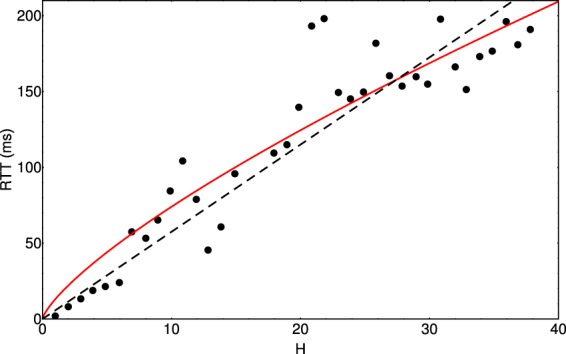
Averaged RTT for different hops number determined from the “traceroute” experiment. The continuous line suggests a power-law fit with an exponent 3/4. The coefficient of determination for such a fit is ${R}^{2}=0.98$. The dashed line indicates a simple linear proportionality, and for such a fit one would get ${R}^{2}=0.96$. The origin locations for the traceroute experiments are in the depicted region (Fig. 1b), while target routers were spread all over the world.

**Figure 10:**
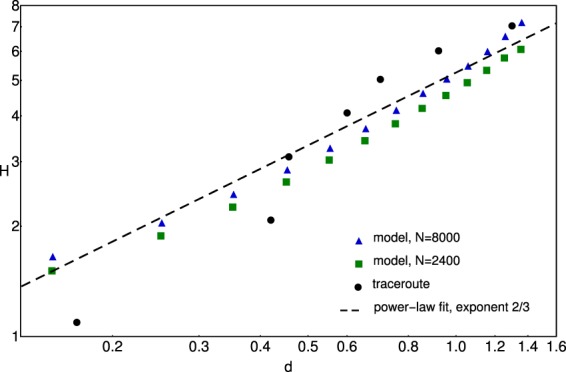
Hop number as a function of geodesic distance. The data extracted from the traceroute experiments on the delimited geographical region is plotted with black dots. The results offered by the model is plotted with green squares ($N=2400$) and blue triangles ($N=8000$). The dashed line indicates a power-law trend with exponent 2/3. The coefficient of determination for such a fit ($y=a\cdot {x}^{2/3}$) is ${R}^{2}=0.98$ for the traceroute data and it is over ${R}^{2}=0.99$ for the datapoints provided by the model. The geodesic distances for the subnetwork revealed by the traceroute experiments is rescaled on the (0, 1) × (0, 1) square.

The observed scaling can be associated with the routing properties of the Internet. The largest contribution to RTT is due to the waiting times encountered at the routers, so one can assume that the measured mean RTT should increase with the number of encountered routers (hops) *H*, up to the target. Indeed, the experimental traceroute results suggests that ${\rm{RTT}}\propto {H}^{\gamma }$ (Fig. 9) with $\gamma \approx 3$/4. By considering $\gamma =3$/4, the statistics of the fit gives a coefficient of determination ${R}^{2}=0.98$. In case one would assume a linear dependence between the RTT and the number of hops ($\gamma =1$) the fit would be less performant, and the best coefficient of determination achieved is ${R}^{2}=0.96$. If one would search also for the best *γ* coefficient which maximizes *R*^2^, we would get $\gamma =0.73$ which would increase the value of *R*^2^ with less than 0.001. In such manner, for the sake of simplicity, the $\gamma =3$/$4=0.75$ choice is justified. As a result, we can also imply that the number of encountered hops is scaling with the distance, the scaling exponent being: (1/2)/(3/4) = 2/3. Results plotted on Fig. 10 suggest the validity of such scaling. For the traceroute data the coefficient of determination for such a regression is ${R}^{2}=0.98$. The fact that the average RTT values are not directly proportional with the *H* values suggests that a simple delay that is in average constant on all routers cannot completely account for the observed ${\rm{RTT}}\propto {d}^{1/2}$ scaling.

For the elaborated model one can study the hops number versus geodesic distance. By using the Breadth-first search algorithm implemented in Igraph’s Python package^31^ one can determine the shortest topological path between the nodes. Since the ICMP echo request protocol is programmed to go on routes with the minimal number of hops, we can assume that any message will follow the shortest topological path. In such manner one can study again the scaling for the number hops (*H*) as a function of the distance between the nodes. Results averaged on several realizations with $N=2400$ and $N=8000$ ($\beta =0.4$) values are presented in comparison with the experimental ones on Fig. 10. One can realize, that both the experimentally observed trend and the one given by the model is a power-law, consistent with an exponent 2/3 and visibly different from 1/2. The trend similar to the experimental results suggests that this simple model captures the origin of the non-trivial scaling between the travel time and geodesic distance.

## Conclusions

From comparison between the model and experimental data we conclude that our simple one parameter wiring model implemented in a geometric space is capable of qualitatively reproducing the observed statistical features of the Internet network at router level. In this view the nontrivial scaling for the average travel time of a message as a function of the geodesic distance is the result of the specific network topology. However, the difference between the scaling exponent for the number of hops as a function of distance (≈2/3), and for the scaling exponent of RTT as a function of distance (≈1/2) suggests that a constant average delay on routers cannot account totally for this scaling. Apart of network topology presumably other elements has to be taken into account for building a more realistic model. This is somehow similar with what we have learned in^18^ when the nontrivial scaling of the human travel time as a function of the geodesic distance was investigated.

## Data Availability

The datasets generated/analyzed during the current study (others than the ones that are publicly available on the indicated links) are available in the FIGHSAHRE repository under the link: https://figshare.com/s/ce595c23e93aad2d7f14.
